# Spectrum of DNA Variants Underlying Deafness in an Ecuadorian Cohort

**DOI:** 10.1007/s10528-026-11319-z

**Published:** 2026-01-10

**Authors:** Anghela Reinoso-Castillo, Memoona Ramzan, Andrea Carrera-Gonzalez, Christian Rivas-Iglesias, Stefanny Montufar, Rodrigo Vinueza-Gavilanes, Carson Smith, Arianne Llamos-Paneque, Mustafa Tekin

**Affiliations:** 1 Universidad Regional Amazónica Ikiam, Parroquia Muyuna km 7 vía Alto Tena, Tena Napo, Ecuador; 2 John P. Hussman Institute for Human Genomics University of Miami Miller School of Medicine, 1501 NW 10th Avenue, BRB-610 (M860), Miami, FL 33136 USA; 3 Molecular Biology and Biochemistry Lab, Universidad Regional Amazónica Ikiam, Parroquia Muyuna km 7 vía Alto Tena, Napo, Tena Ecuador; 4 Hospital de Especialidades Fuerzas Armadas No.1 Quito-Ecuador, Ecuador; 5 Center for Applied Medical Research (CIMA) Gene Therapy and Regulation of Gene Expression Program, University of Navarra, Pamplona, Spain; 6 Israel Technological University School of Medicine, Quito, Ecuador; 7 Facultad de Ciencias Médicas Escuela de Odontología, Universidad Internacional del Ecuador (UIDE), de la Salud y de La Vida, Quito, Pichincha Ecuador; 8 Dr. John T. Macdonald Foundation Department of Human Genetics, University of Miami Miller School of Medicine, 1501 NW 10th Avenue, BRB-610 (M860), Miami, FL 33136 USA

## Abstract

The genetic etiology of hearing loss (HL) in Ecuador remains largely unexplored. This study investigates the spectrum of genetic variants associated with HL in a cohort of 66 Ecuadorian families using Exome or Genome sequencing (ES/GS). We identified pathogenic and likely pathogenic variants underlying HL in 27 families (41%). While variants were detected in 15 different genes, only *GJB2* (in 12 families) and *TMC1* (in 3 families) variants were identified in more than one family. The NM_004004.6 (*GJB2*):c.19 C > T (p.Gln7*) and NM_004004.6 (*GJB2*):c.35delG (p.Gly12Valfs*2) variants were more common than other alleles in *GJB2*. In some families, we detected variants of uncertain significance (VUS) in well-established HL genes and classified them as “possibly solved” due to their rarity and equivocal functional predictions. This study provides valuable insights into the genetic basis of HL in Ecuador and lays the groundwork for improved regional genetic diagnostics and management.

**Graphical Abstract:**

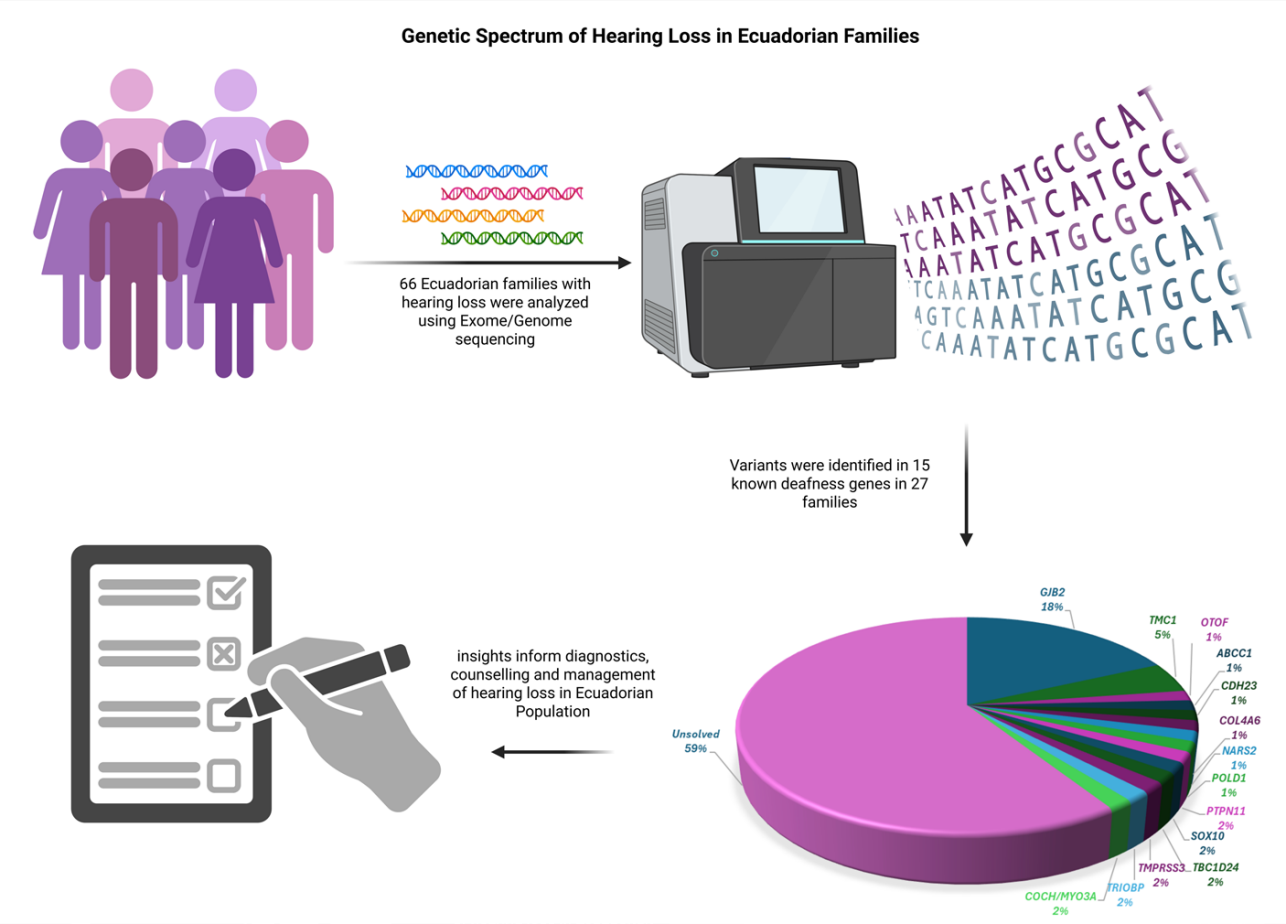

**Supplementary Information:**

The online version contains supplementary material available at 10.1007/s10528-026-11319-z.

## Introduction

Hearing loss (HL) is a common sensory disorder affecting over 400 million people globally. (Davis and Hoffman 2019; Walls et al. 2020). In developed countries, it is estimated that one to three out of every 1,000 newborns have some degree of hearing impairment, while this rate can reach 6 per 1000 in developing nations. (Morton and Nance 2006; Stringer 2017). HL is typically classified based on standard audiometric values as mild (26–40 dB), moderate (41–60 dB), severe (61–80 dB), or profound (> 81 dB) (Koffler et al. 2015).

HL can have genetic or environmental origins worldwide, with genetic factors accounting for approximately 60% of cases. Genetic HL can be further categorized into two types: non-syndromic HL (NSHL), which is characterized by the absence of other abnormalities, and syndromic HL (SHL), which is accompanied by various additional findings. NSHL is the most common type, representing 70% of genetic HL, and can be inherited in an autosomal recessive (75–80%), autosomal dominant (20%), X-linked (< 2%), or mitochondrial (< 1%) manner (Koffler et al. 2015).

*GJB2* is the most reported gene associated with autosomal recessive nonsyndromic hearing loss (ARNSHL) in many populations, although not in all. It encodes connexin 26, a crucial component of gap junctions in the cochlea, which are essential for hearing. Since *GJB2* is a conserved single exon gene, many nucleotide changes lead to causal variants. Over 400 pathogenic variants have been documented in *GJB2* (https://www.hgmd.cf.ac.uk/ac/index.php) (Kelsell et al. 1997; Duman and Tekin 2012; Chan and Chang 2014), reflecting its high allelic heterogeneity. Additionally, recurring founder mutations such as NM_004004.6 (*GJB2*):c.35delG (p.Gly12Valfs*2) in Europeans, NM_004004.6 (*GJB2*):c.235delC (p.Leu79Cysfs*3) in East Asians, and NM_004004.6 (*GJB2*):c.167delT (p.Leu56Argfs*26) in Ashkenazi Jews contribute to the wide allelic diversity seen worldwide. Its frequent inclusion in HL diagnostic panels has also enabled the identification of many rare, pathogenic variants worldwide.

In Ecuador, HL is a significant public health concern, with affected individuals representing 14.12% of the population registered with the Consejo Nacional para la Igualdad de Discapacidades (CONADIS 2019). However, the relationship between different gene mutations and the epidemiology of HL in Ecuador remains largely unknown. This study aims to determine the prevalence of genetic variants associated with deafness in an Ecuadorian population, providing valuable insights into the genetic landscape of HL in this country.

## Materials and Methods

### Ethical Considerations

The Institutional Review Board at the University of Miami, USA (Protocol# 20081138) and the Bioethics Committee from Hospital de Especialidades Fuerzas Armadas No. 1 Quito-Ecuador (HE FF.AA No.1) (Ecuador) approved this study. Patients or their parents/legal guardians signed the informed consent form.

## Patients and Clinical Analysis

This study was carried out in collaboration with the Servicio de Genética Médica of HE FF.AA No.1 in Quito-Ecuador, and the University of Miami, USA. We included 66 Ecuadorian families with genetic HL after careful questioning about familial history and audiological examination. Audiometry was performed under ambient noise conditions, and pure tone thresholds were measured at 0.25, 0.5, 1, 2, 4, and 8 kHz. The hearing assessment of toddlers and children under 8 years was conducted using play audiometry. Romberg and tandem gait tests (Fregly and Graybiel 1970; Rogers 1980) were performed to evaluate vestibular function. Detailed questioning by the clinical geneticist regarding family history, disease onset and progression, kinship, use of ototoxic medication, medical history for heart and renal dysfunction, skin, eye, or abnormal hair pigmentation, night blindness, and trauma was completed to check the suitability of the family for inclusion in the study. The inclusion criteria were sensorineural deafness proven by audiometry, Ecuadorian nationality, and signing informed consent about the study.

## Genetic Analysis

DNA was isolated from 4 mL of peripheral blood from affected probands and available family members using the High Pure PCR Template Preparation Kit (Roche Diagnostics GmbH, Mannheim, Germany) according to the manufacturer´s protocol. All probands were analyzed by Sanger sequencing to detect variants in *GJB2*, a common cause of hearing loss. Exome sequencing (ES) was performed on the DNA of 54 probands who were negative for *GJB2* variants, and Genome sequencing (GS) was performed on five cases where ES did not identify a causal variant, following previously reported protocols (Ramzan et al. 2023). After Exome and Genome sequencing, the obtained data were aligned with the human reference genome assembly (GRCh37/hg19, UCSC) using the Burrows-Wheeler Aligner (http://bio-bwa.sourceforge.net), and variant calling was performed using FreeBayes (https://github.com/freebayes/freebayes). Briefly, all the known deafness genes were analyzed for the presence of single nucleotide or indel variants. All samples were processed using the defaut institutional pipeline to ensure uniformity with previously sequenced cases and to avoid batch effects introduced by re-alignment or re-calling on a different reference genome. Noncoding, intergenic, and structural variants were examined in the data after GS. Copy Number Variants (CNVs) were assessed using CNVnator (Abyzov et al. 2011) and CoNIFER v.02.2 (Krumm et al. 2012) with default parameters. Data were reanalyzed to determine the presence of any candidate variant if it remained negative after the preliminary analysis. Variants were interpreted using the American College of Medical Genetics and ClinGen HL expert panel guidelines (Richards et al. 2015; Oza et al. 2018; Tavtigian et al. 2020). Each applied code is shown in Table S1.

For each variant, we evaluated population frequency (gnomAD), conservation, in silico prediction scores (REVEL, AlphaMissense, MaxEntScan, SpliceAI), functional impact, and literature evidence. Where applicable, segregation and phenotype correlation were also considered. The term “possibly solved” was applied in families where the variant was classified as a variant of uncertain significance (VUS) by ACMG criteria but had supportive evidence such as segregation with the disease, expression of the gene in the relevant tissue, gene-disease association supported by literature, or a plausible mechanism of pathogenicity based on in silico tools and conservation. Sanger sequencing was performed to confirm the co-segregation of the variant with the phenotype in the respective proband and family.

## Results

### Patients’ Characteristics

The project enrolled 66 families (49 simplex and 17 multiplex), totaling 80 affected and 105 unaffected individuals. All the affected individuals had bilateral, congenital, or prelingual HL with a severe to profound degree of HL, and no additional clinical symptoms were identified at the time of enrollment. All participating parents of the respective probands had normal hearing, except for families 1 and 27. The participating affected parents in these families had no reported history of HL evident after detailed questioning.

### Genetic Variants

**Fig. 1:**
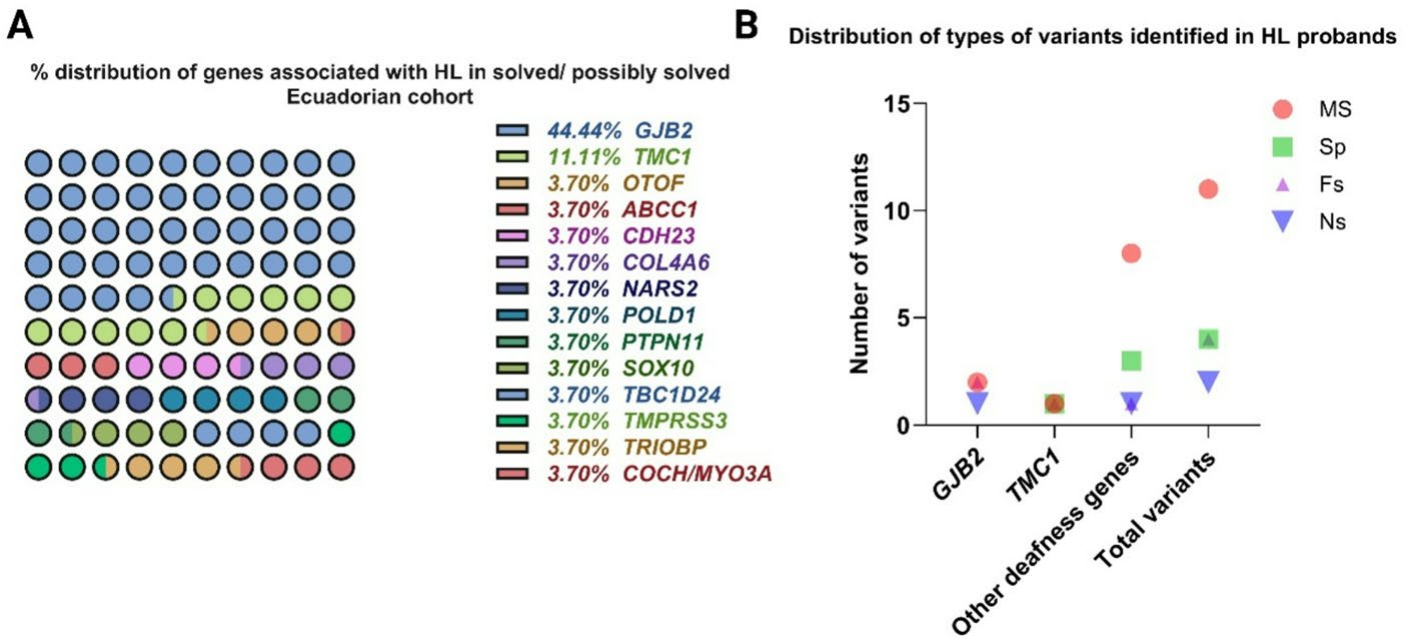
Overview of the genetic findings in the Ecuadorian hearing loss (HL) cohort. **A** Percentage distribution of genes associated with HL in solved/possibly solved Ecuadorian cases after Sanger sequencing and exome data analysis. A waffle chart represents the proportion of genes contributing to solved or possibly solved HL cases. Each circle represents a percentage of the cohort with an identified variant in a specific gene. The most frequently implicated gene is *GJB2* (44.44%), followed by *TMC1* (11.11%). Several other genes, including *OTOF*,* ABCC1*,* CDH23*,* COL4A6*,* NARS2*,* POLD1*,* PTPN11*,* SOX10*,* TBC1D24*,* TMPRSS3*,* TRIOBP*, and *COCH/MYO3A*, each contribute 3.70% to the solved cases. The color-coded labels correspond to each gene, aiding in the visual differentiation of their respective contributions. **B** Distribution of types of variants associated with HL. A scatter plot depicts the number and types of genetic variants identified in different deafness-associated genes. The x-axis represents the genes along with the total number of variants identified. The y-axis denotes the number of variants per category. The color-coded markers indicate different types of variants: missense (MS, red circles), splice site (Sp, green squares), frameshift (Fs, purple triangles), and nonsense (Ns, blue inverted triangles). This distribution provides insights into the variant landscape among the studied cohort

A genetic diagnosis was reached in 27 families out of 66 (41%) after screening via Sanger sequencing and ES (Table 1 and Table S2). Twenty-one variants were identified in 15 genes associated with HL (Table 1). No pathogenic or likely pathogenic CNVs relevant to hearing loss were identified. Among these solved families, we identified two recurrent NM_004004.6 (*GJB2*): c.35delG (p.Gly12Valfs*2), NM_004004.6 (*GJB2*): c.19 C > T (p.Gln7*) and three unique variants (NM_004004.6 (*GJB2*): c.596 C > T (p.Ser199Phe), NM_004004.6 (*GJB2*): c.645delT (p.Arg216Aspfs*18), and NM_004004.6 (*GJB2*): c.427 C > T (p.p.Arg143Trp) in *GJB2* in 12 unrelated probands after Sanger sequencing (Figure S1, Table S3). Fifty-four probands underwent ES, and five were subjected to GS as an additional test to find the cause of HL. We identified 16 variants in 15 probands after ES in known deafness genes, including *ABCC1*, *CDH23*, *COL4A6*, *NARS2*, *OTOF*, *TBC1D24*, *TMC1*, *TMPRSS3*, *TRIOBP*, *COCH*, and *MYO3A* (Table 1 and Figure S2). Our findings included causative variants in syndromic HL genes *PTPN11*, *SOX10*, and *POLD1* (Fig. 1A) despite clinical assessment indicating no syndromic features at the time of study enrollment. A splice VUS in *POLD1* identified in our Ecuadorian cohort may exhibit variable penetrance and expressivity. In other patients, *POLD1* mutations are typically associated with Mandibular hypoplasia, deafness, progeroid features, and lipodystrophy (MDPL) syndrome (Shastry et al. 2010; Weedon et al. 2013).

**Table 1:** Detail of variants identified in participating hearing loss families after ES

Family ID	Gene	DFNB/A/X	OMIM ID	Transcript/ variant (hg19)	Variant effect/ Zyg	Global AF	FAF95 PopMax	AFR AF	AMR AF	EAS AF	SAS AF	NFE AF	ASJ AF	FIN AF	RMI AF	Pathogenicity scores		ACMG criteria	ACMG classification	PMID
																REVEL	AM			
Family 1	*ABCC1*	DFNA77	158,343	NM_004996.4 c.1769 A > C p.Asn590Thr	Ms/Het	Absent	Absent	Absent	Absent	Absent	Absent	Absent	Absent	Absent	Absent	0.3	0.59	PM2_sup, PM5	VUS	31,273,342
Family 2	*CDH23*	DFNB12	605,516	NM_022124.5 c.1099 C > T p.Leu367Phe	Ms/Hom	4e06	0	0	0	0	3e05	0	0	0	0	0.3	n/a	PM2_mod	VUS	n/a
Family 3	*COL4A6*	DFNX6	300,914	NM_001847.3 c.903_904delCT p.Leu302Metfs*27	Fs/Hemi	5e06	n/a	n/a	n/a	n/a	n/a	n/a	n/a	n/a	n/a	n/a	n/a	PM2_mod	VUS	n/a
Family 16	*NARS2*	DFNB94	612,803	NM_024678.6 c.451 C > T p.Val151Ile	Ms/Hom	2e05	1e05	8e05	0	0	0	3e05	0	0	0	0.09	0.08	PM2_mod, PP1_sup	VUS	n/a
Family 17	*OTOF*	DFNB9	603,681	NM_194248.3 c.4227 + 1G > T	Sp/Hom	8e06	0	0	2e05	0	0	8e06	0	0	0	n/a	n/a	PM2_mod, PVS1_strong, PP1_sup, PS4_sup	LP	18,381,613
Family 18	*POLD1*	n/a	174,761	NM_002691.4 c.1138–3 C > A	Sp/Het	Absent	Absent	Absent	Absent	Absent	Absent	Absent	Absent	Absent	Absent	n/a	n/a	PM2_mod, PP3_mod	VUS	n/a
Family 19	*PTPN11*	n/a	176,876	NM_002834.5 c.417G > C p.Glu139Asp	Ms/Het	Absent	Absent	Absent	Absent	Absent	Absent	Absent	Absent	Absent	Absent	0.7	0.9	PM2_mod, PP3_mod, PS4_mod	LP	11,992,261
Family 20	*SOX10*	n/a	602,229	NM_006941.4 c.378 C > A p.Tyr126*	Ns/Het	Absent	Absent	Absent	Absent	Absent	Absent	Absent	Absent	Absent	Absent	n/a	n/a	PM2_mod, PVS1_strong,	LP	33,442,024
Family 21	*TBC1D24*	DFNB86/DFNA65	613,577	NM_001199107.2 c.1321 C > T p.Arg441Cys	Ms/Hom	1e05	7e06	0	0	5e05	0	2e05	0	0	0	0.3	0.3	PM2_sup	VUS	n/a
Family 22	*TMC1*	DFNB7/11/DFNA36	600,974	NM_138691.3 c.2130-1delG	Sp/Hom	Absent	Absent	Absent	Absent	Absent	Absent	Absent	Absent	Absent	Absent	n/a	n/a	PM2_mod, PVS1_strong, PS4_sup	LP	11,850,618
Family 23	*TMC1*	DFNB7/11/DFNA36	600,974	NM_138691.3 c.1304 C > T p.Pro435Leu	Ms/Hom	Absent	Absent	Absent	Absent	Absent	Absent	Absent	Absent	Absent	Absent	0.6	0.9	PM2_mod	VUS	n/a
Family 24	*TMC1*	DFNB7/11/DFNA36	600,974	NM_138691.3 c.15delA p.Val6Tyrfs*33	Fs/Hom	Absent	Absent	Absent	Absent	Absent	Absent	Absent	Absent	Absent	Absent	n/a	n/a	PM2_mod, PVS1_strong	LP	n/a
Family 25	*TMPRSS3*	DFNB8/10	605,511	NM_001256317.3 c.782 + 1G > A	Sp/Hom	7e06	3e06	0	0	0	0	7e06	0	0	0	n/a	n/a	PM2_mod, PVS1_strong	VUS	n/a
Family 26	*TRIOBP*	DFNB28	609,761	NM_001039141.3 c.5752G > A p.Ala1918Thr	Ms/Hom	Absent	Absent	Absent	Absent	Absent	Absent	Absent	Absent	Absent	Absent	0.04	0.07	PM2_mod	VUS	n/a
Family 27	*COCH*	DFNB110/DFNA9	603,196	NM_004086.3 c.1436 A > G p.Tyr479Cys	Ms/Het	Absent	Absent	Absent	Absent	Absent	Absent	Absent	Absent	Absent	Absent	0.8	0.9	PM2_mod, PP3_mod	VUS	n/a
	*MYO3A*	DFNB30/DFNA90	606,808	NM_017433.5 c.2951G > A p.Arg984Gln	Ms/Het	2e05	1e05	0	2e05	0	6e05	2e05	0	0	0	0.8	0.4	PM2_mod, PP3_mod	VUS	n/a

All the frequencies were observed in gnomADv2.1.1, GRCh37/hg19

*OMIM* online mendelian inheritance in man, *zyg* Zygosity, *Ms* missense, *Ns* nonsense, *Fs* frameshift, *Sp* splicing, *af *allele frequency, *afr* African/African American, *ami* amish, *amr* admixed American, *asj* ashkenazi jewish, *eas* East Asian, *fin* Finnish, *nfe* nonFinnish European, *sas* South Asian, *rmi* remaining individuals”, *n/a* not applicable, *REVEL* rare exome variant ensemble learner, *AM* AlphaMissense, *sup* supportive, *mod* moderate, *VUS* variant of uncertain significance, *LP* likely pathogenic, *P* pathogenic, *Hom* homozygous, *Het* heterozygous, *N/A* not available

In total, 11 are missense variants, and the rest are nonsense, frameshift, and splice variants (Fig. 1B). All variants identified after exome sequencing are unique to each proband and co-segregated with the condition. Eight of these identified variants in known genes are being reported for the first time for causing HL (Table 1). All the probands were homozygous for the detailed variants in Table 1 except those in *PTPN11*, *COCH*, *MYO3A*, *ABCC1*, *POLD1*, and *SOX10*. As these samples were obtained 15–20 years ago, some detailed pedigree records, such as the presence or absence of consanguinity, are no longer accessible; however, all available family information was reviewed and incorporated where possible. The observed homozygosity in the presented pedigrees most likely reflects population isolation rather than consanguinity.

The splicing prediction tools (SpliceAI, HumanSpliceFinder (HSF), MaxEntScan, and varSEAK) used to estimate the effects of four splicing variants in *OTOF*, *POLD1*, *TMC1*, and *TMPRSS3* showed that all can disrupt authentic splice sites, resulting in truncated proteins. NM_194248.3 (*OTOF*): c.4227 + 1G > T disrupts the canonical donor splice site at exon 34. SpliceAI predicts complete donor loss (Δ = 1.00) and generation of a nearby cryptic donor at + 2 (Δ = 0.48). The scores emphasize the likely usage of + 2 cryptic donor, resulting in retention of 2 intronic nucleotides, a frameshift at the exon 34 junction, leading to a premature termination codon within the subsequent coding sequence. Exon 34 skipping is an alternative but less favored mechanism; however, it would likewise cause a frameshift.

**NM_002691.4** (*POLD1*) c.1138–3 C > A lies at the − 3 position of the 3′ acceptor. HSF/MaxEntScan show a ~ 25% drop in acceptor strength (MaxEntScan 9.85 → 6.01), and varSEAK classifies it as Class 5 (splicing effect), predicting loss of the authentic acceptor with selection of a cryptic acceptor ~ 41 bp into the exon. This would delete 41 bp at the 5′ end of the exon, causing a frameshift and premature termination. SpliceAI does not flag this variant (Δ = 0.00), however, motif-based tools such as HSF, MaxEntScan, and varSEAK consistently indicated disruption of the canonical acceptor and activation of a nearby cryptic site, supporting an aberrant splicing event likely resulting in loss of function.

**The NM_138691.3** (*TMC1*) c.2130-1delG affects the “AG” acceptor preceding exon 22. SpliceAI predicts a complete loss of the canonical acceptor site (Δ = 1.00) and activation of a cryptic acceptor 36 bp downstream (Δ = 0.60). This may lead to skipping of 36 bp (12 codons) at the start of exon 22, resulting in an in-frame deletion of 12 amino acids (or, less likely, exon 22 skipping and a frameshift).

**NM_001256317.3** (*TMPRSS3*) c.782 + 1G > A disrupts the donor splice site of exon 8. SpliceAI predicts donor loss (Δ = 0.58) with no nearby meaningful cryptic donor. Modeling with the provided exon sequences indicates skipping of exon 8, resulting in a frameshift and a premature termination within exon 9 after 44 codons.

No variant in known deafness genes was identified in 5 families tested by GS. The etiology of HL in 39 (59%) families remained unresolved after ES/GS, as no candidate causal gene or mutation was identified. We analyzed unsolved families using all genes/variants (common and rare) in the OMIM database, and none showed HL linked to a known deafness gene.

Family 27 is a trio that includes the proband and parents with HL. Upon genetic analysis, we identified two variants in *MYO3A* and *COCH*, both of which are known to cause autosomal recessive and dominant forms of HL. When we tested these two genes for segregation, the mother was homozygous for the *MYO3A* variant c.2951G > A, and the father was homozygous for the c.1436 A > G variant in *COCH*. However, the proband was heterozygous for the two identified variants. There was insufficient data available on the age of onset and progression of HL in parents. At the same time, the proband had profound HL at age 8, which differed from the reported autosomal dominant phenotype (mild or moderate to severe HL) due to either of the genes (Robertson et al. 1998; Dantas et al. 2018).

ACMG criteria were applied to classify the variants, yielding 10 pathogenic/likely pathogenic variants and 11 VUS (Table 1 and Table S3). In 17/27 cases, a pathogenic or likely pathogenic variant was identified and segregated. 10/27 cases were categorized as potentially solved, in which a VUS was segregated with a known deafness gene. When including only pathogenic or likely pathogenic variants, the diagnostic yield was 27%. Including cases with VUS in known HL genes that met high-interest criteria increased the yield to 41%; however, these were marked as “possibly solved” and were interpreted with caution.

## Discussion

The genetic makeup of the Ecuadorian population predominantly carries indigenous genetic contributions (approximately 60–70%), accompanied by European (~ 20–30%) and some African (~ 5–10%) ancestries (Bryc et al. 2010). Such complex admixture has significantly influenced the susceptibility and manifestation of hereditary disorders, including hearing impairment in Ecuador.

Our study identified genetic variants associated with non-syndromic and syndromic forms of HL. The detection of recurrent pathogenic variants such as the NM_004004.6 (*GJB2*): c.35delG (p.Gly12Valfs*2), and NM_004004.6 (*GJB2*): c.19 C > T (p.Gln7*) variants in *GJB2* is similar to the findings in other Latin American populations (Figueroa-Ildefonso et al. 2019; Bouzaher et al. 2020; Lezirovitz and Mingroni-Netto 2022). A study on more than 100 subjects concluded that NM_004004.6 (*GJB2*): c.19 C > T (p.Gln7*) is this population’s predominant cause of HL (Paz-y-Mino et al. 2014).

Majority of HL-linked genes, such as *GJB2* and *TBC1D24*, are inherited in a recessive manner, but heterozygous variants can influence the condition through various mechanisms. Our study found heterozygous variants in both dominant and recessive genes. For dominant or syndromic genes such as *ABCC1*, *COCH*, *MYO3A*, *SOX10*, *PTPN11*, and *POLD1*, segregation analysis showed that these variants co-segregated with HL, supporting their potential pathogenicity rather than being benign variants. The heterozygous variants may act via dominant-negative effects, disrupting normal protein function, or via haploinsufficiency, in which one allele is insufficient for normal function (Gerasimavicius et al. 2022). Overall, the findings in this study highlight the genetic and mechanistic complexity of HL, in which even a single heterozygous variant can have functional effects depending on the gene, variant, and inheritance pattern.

The variants identified in this cohort across all genes converge on a few fundamental cochlear processes, including ionic coupling, mechanoelectrical transduction, synaptic vesicle release, and regulation of the extracellular matrix and signaling. The identified variants are in conserved regions or key motifs of cochlear proteins. For instance, *GJB2* frameshift and nonsense variants may truncate connexin 26, abolishing gap-junction channels that recycle K⁺ through supporting cells and may collapse the endocochlear potential (Choi et al. 2011; Wingard and Zhao 2015). *TMC1* variants may disrupt transmembrane domains required for the hair-cell mechanotransduction channel (Kurima et al. 2002; Kawashima et al. 2015). Similarly, missense changes in *TRIOBP and MYO3A* can affect actin bundling (Kitajiri et al. 2010) and motor regions critical for stereocilia stability (Gunther et al. 2022). The splice-site variant in *OTOF* can cause the removal of C2-domain exons essential for Ca²⁺-triggered vesicle exocytosis (Michalski et al. 2017), and the missense substitution in the vWFA2 domain in COCH likely causes protein misfolding and matrix aggregation in the spiral ligament (Cho et al. 2012). For VUS in genes such as *CDH23*, *TBC1D24*, *ABCC1*, *NARS2*, *COL4A6*, *PTPN11*, *SOX10*, and *POLD1*, predicted effects from different mutation analysis tools indicate disrupted structural or catalytic domains and aberrant splicing. Therefore, even VUS can plausibly impair ionic homeostasis, mechanotransduction, synaptic signaling, or matrix composition, each of which is sufficient to cause HL. Future functional assays examining mRNA abundance, protein localization, and domain stability will help validate these mechanisms.

The diagnostic yield based strictly on pathogenic and likely pathogenic variants in our cohort was 27%, falling slightly below the range typically observed in similar cohorts. Upon the inclusion of ‘possibly solved’ families, defined as VUS with supporting evidence such as co-segregation, conservation, and deleterious in silico predictions, the overall yield increased to 41% with 17 of 49 simplex (34.7%) and 10 of 17 multiplex (58.8%) families receiving a molecular diagnosis. This solved rate is comparable to other published studies reporting diagnostic yields of 30–50% in mixed and consanguineous populations (Shearer and Smith 2015; Sloan-Heggen et al. 2016; Adeyemo et al. 2022; Ramzan et al. 2023). This convergence across populations likely reflects shared technical and interpretive constraints rather than population-specific factors. A substantial proportion of genetic variation lies in noncoding, structural, or complex regions. Moreover, limited gene discovery and functional evidence also hinder the classification of VUS. Consequently, the diagnostic yield remains at about 40–45% in Mendelian disease cohorts despite advances in sequencing technologies and variant databases.

Variant curation is an evolving process that is continually refined by advances in ACMG guidelines and collaborative data sharing. Our study has classified certain families as ‘possibly solved’ based on rare, highly conserved variants in well-established deafness genes with high pathogenicity scores. However, due to the current lack of functional data or additional reported cases, these variants remain classified as VUS rather than being upgraded to likely pathogenic or pathogenic. Over time, as more cases with the same variants are identified and additional evidence emerges, their role in disease association may become more evident, leading to a more definitive classification.

Our study provides clinically valuable insights into the genetic etiology of HL in Ecuador, identifying mutations in both globally prevalent genes and rare or syndromic-associated genes. The complex genetic etiology in the Ecuadorian population reflects intrinsic complexities of Latin American genetics, underscoring the need for expanded research and enhanced clinical follow-up for affected individuals. Additionally, the unsolved families after ES and/or GS can be an excellent source for identifying new candidate variants or genes. For the unresolved cases in this cohort, several complementary approaches may improve diagnostic yield. Re-analysis of existing sequencing data using updated databases and long-read sequencing could help identify structural variants, complex indels, and other alleles that are not reliably captured by short-read platforms. Functional characterization of promising candidate genes, particularly those with expression or biochemical relevance to inner-ear pathways, may provide further insight into their potential role in hearing loss. These combined strategies outline a feasible framework for resolving the remaining unsolved cases. Ultimately, these findings pave the way for implementing more comprehensive genetic screening and improved diagnosis and management of HL in Ecuador and similar Latin American regions.

## Limitations and Future Studies

While this study advances our understanding of the genetic basis of hearing loss in the Ecuadorian population, several aspects could be addressed in future research. Functional validation of VUS by incorporating in vitro assays or computational modeling could aid in clarifying their pathogenicity. Although clinical assessments were conducted at enrollment, more systematic evaluations and longitudinal follow-up may help uncover subtle or age-dependent syndromic features. Global ancestry estimation was not performed, but could contextualize variant interpretation and help resolve unsolved cases in this admixed population. Additionally, limited data on age of onset, disease progression, and family structure constrained genotype-phenotype correlation and segregation analysis. Future studies with larger cohorts, deeper genomic analysis including noncoding regions and novel gene discovery, and standardized phenotypic documentation will be key to improving diagnostic yield in underrepresented populations.

## Supplementary Information

Below is the link to the electronic supplementary material.

Supplementary Material 1

## Data Availability

All data generated or analyzed during this study are submitted to NCBI’s Bioproject with submission ID SUB15879724.
