# Supplementary material for: Spectrum of DNA Variants Underlying Deafness in an Ecuadorian Cohort
Source: Biochem Genet. 2026 Jan 10;64(4):5792–806. doi: 10.1007/s10528-026-11319-z (PMC13388514; doi:10.1007/s10528-026-11319-z)
Supplement: Supplementary file 1 — Supplementary Material 1 [file 10528_2026_11319_MOESM1_ESM.docx]

**Spectrum of DNA variants underlying deafness in an Ecuadorian cohort**

Anghela Reinoso-Castillo^1^, Memoona Ramzan^2^, Andrea Carrera-Gonzalez^3^, Christian Rivas-Iglesias^4^, Stefanny Montufar^4^, Rodrigo Vinueza-Gavilanes^5^, Carson Smith^2^, Arianne Llamos-Paneque^4,6^, Mustafa Tekin^2,7*^

^1^Universidad Regional Amazónica Ikiam, Parroquia Muyuna km 7 vía Alto Tena, Tena, Napo, Ecuador.

^2^John P. Hussman Institute for Human Genomics, University of Miami Miller School of Medicine, Miami, FL, USA

^3^ Molecular Biology and Biochemistry Lab, Universidad Regional Amazónica Ikiam, Parroquia Muyuna km 7 vía Alto Tena, Tena, Napo, Ecuador

^4^Hospital de Especialidades Fuerzas Armadas No. 1 Quito-Ecuador

^5^Gene Therapy and Regulation of Gene Expression Program, Center for Applied Medical Research (CIMA), University of Navarra, Pamplona, Spain

^6^Facultad de Ciencias Médicas, de la Salud y de La Vida, Escuela de Odontología, Universidad Internacional del Ecuador (UIDE). Quito, Pichincha, Ecuador

^7^Dr. John T. Macdonald Foundation Department of Human Genetics, University of Miami Miller School of Medicine, Miami, FL, US

^*^**Corresponding author:**

Mustafa Tekin

1501 NW 10th Avenue, BRB-610 (M860)

Miami, FL 33136

Phone: +1 (305)243-2381

E-mail: mtekin@med.miami.edu


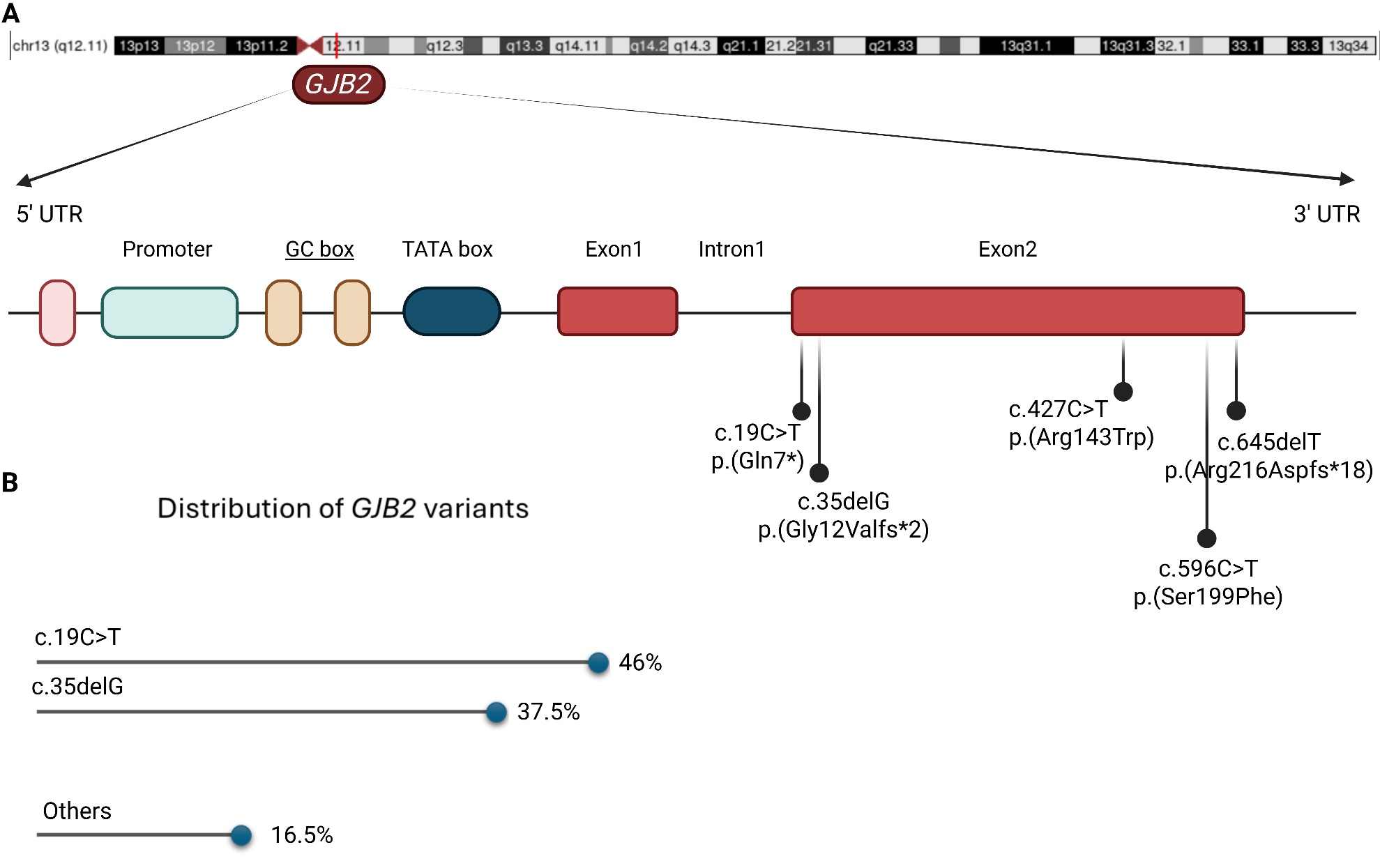


**Figure S1: Schematic representation of *GJB2* and distribution of identified alleles. (A)** Chromosomal location of *GJB2* and essential domains. Four different variants were identified in exon 2. **(B)**The lollipop chart represents the frequency of commonly identified *GJB2* alleles among 12 probands tested by Sanger sequencing. A total of 24 alleles were identified, and the most frequent variant was c.19C>T, accounting for 46% of the alleles, followed by c.35delG at 37.5%. Other *GJB2* variants collectively comprised 16.5% of the detected alleles.


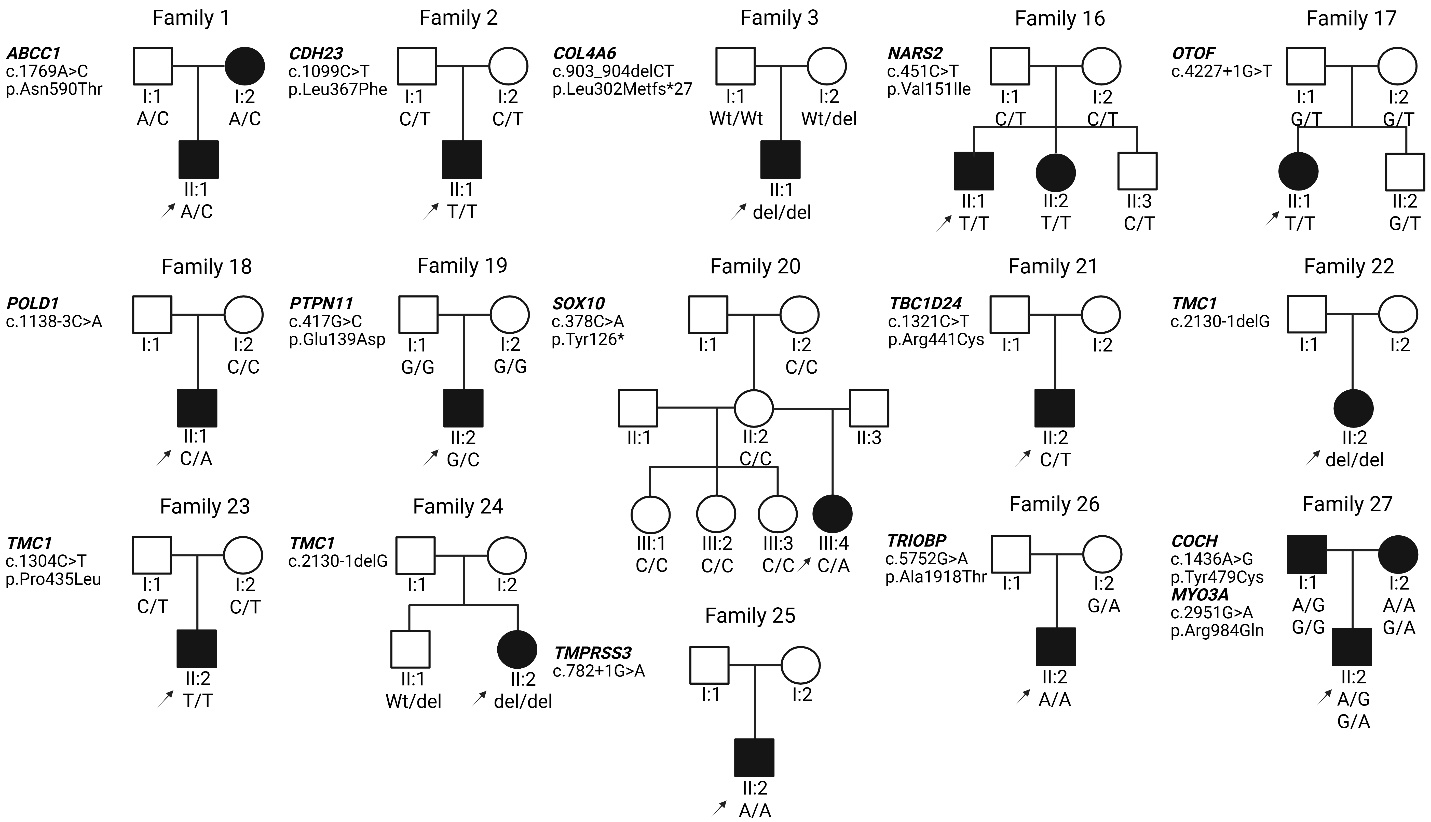


**Figure S2:** Pedigree diagram of families showing the variant detail and segregation with the phenotype.

**Table S1:** **Updated ACMG/AMP criteria used in this study (post‑2019 ClinGen SVI updates)**

| **Code** | **Current definition (2021–2024 guidance)** |
| --- | --- |
| PVS1 (Very strong/Strong/Moderate/Supporting) | Predicted loss‑of‑function variant (nonsense, frameshift, ±1/2 splice, initiation loss, or relevant exon/gene deletion) in a gene where LoF is an established disease mechanism.  Applied as PVS1_strong in our study as per the PVS1 decision‑tree (considering NMD, exon/region, and known mechanism). |
| PS3 / BS3 (Strong/Moderate/Supporting) | Well‑validated functional assay shows a damaging effect (PS3) or no damaging effect (BS3). Strength is set by assay validation (appropriate controls, reproducibility, dynamic range, predefined thresholds) and disease mechanism relevance.  Applied as PS3_moderate and PS3_supporting for the variants where well-validated functional assays were available in literature. |
| PS4 (Strong/Moderate/Supporting) | Case enrichment of the variant in affected individuals vs. controls, using quantitative odds‑ratio/likelihood‑ratio (PS4‑LR) mapped to strength, or disease‑specific VCEP case‑count rules when robust controls are unavailable. |
| PM2_Supporting | Absent or sufficiently rare in well‑curated population datasets (e.g., gnomAD PopMax/FAF95) for the gene/disease model. |
| PM5 | Different missense change at the same amino‑acid residue previously established as pathogenic/likely pathogenic, with consistent mechanism (not truncating LoF). |
| PP1 / BS4 (Strong/Moderate/Supporting) | Co‑segregation (PP1) or non‑segregation (BS4) evaluated with a LOD‑based points framework; cumulative LOD sets Supporting/Moderate/Strong. Consider phenotype specificity (PP4) when interpreting segregation.  Applied as PP1_supporting in our study, where enough evidence of co segregation supported this strength level. |
| PP3 / BP4 | Computational evidence supports a deleterious (PP3) or benign (BP4) effect using pre‑specified tool(s) with validated, gene/disease‑appropriate thresholds. For splicing prediction, MaxEnt scan was used as described and we avoided double‑counting with PVS1. |

**Table S2:** Sequential testing method applied to the participating families and number of solved and unsolved cases.

| Total enrolled families | Study method | Number of participating families | | | Solved cases | | | Unsolved cases | | | Total solved (%) | | | Total unsolved (%) | | |
| --- | --- | --- | --- | --- | --- | --- | --- | --- | --- | --- | --- | --- | --- | --- | --- | --- |
|  |  | Simplex | Multiplex | | Simplex | Multiplex | | Simplex | Multiplex | | Simplex | Multiplex | | Simplex | Multiplex | |
| 66 | Sanger sequencing | 49 | | 17 | 8 | | 4 | 41 | | 13 | 16 | | 24 | 84 | | 76 |
|  | Exome sequencing | 41 | | 13 | 9 | | 6 | 32 | 7 | | 22 | | 46 | 78 | | 54 |
|  | Genome sequencing | 4 | | 1 | 0 | | 0 | 32 | 7 | |  | |  |  | |  |

**Table S3:** Details of variants identified in *GJB2* segregating in hearing loss cases after Sanger sequencing

| **Case ID** | **Gene** | **Transcript/variant**  **(hg19)** | **Variant effect/Zyg** | **Global AF** | **FAF95 PopMax** | **AFR AF** | **AMR AF** | **EAS AF** | **SAS AF** | **NFE AF** | **ASJ AF** | **FIN AF** | **RMI AF** | **Pathogenicity scores** | | **ACMG criteria** | **ACMG classification** | **PMID** |
| --- | --- | --- | --- | --- | --- | --- | --- | --- | --- | --- | --- | --- | --- | --- | --- | --- | --- | --- |
|  |  |  |  |  |  |  |  |  |  |  |  |  |  | **REVEL** | **AM** |  |  |  |
| Case 4 | *GJB2* | NM_004004.6  c.35delG  p.Gly12Valfs*2 | Fs/Hom | 0.006 | 0.008 | 0.001 | 0.004 | 0.0 | 0.0008 | 0.009 | 0.003 | 0.008 | 0.007 | N/A | N/A | PVS1_strong, PS3_mod, PS4_strong | P | 9139825 |
| Case 5 | *GJB2* | NM_004004.6  c.35delG  p.Gly12Valfs*2 | Fs/Hom | 0.006 | 0.008 | 0.001 | 0.004 | 0.0 | 0.0008 | 0.009 | 0.003 | 0.008 | 0.007 | N/A | N/A | PVS1_strong, PS3_mod, PS4_strong | P | 9139825 |
| Case 6 | *GJB2* | NM_004004.6  c.19C>T  p.Gln7* | Ns/Het | 8e06 | 9e06 | 0.0 | 5e04 | 0.0 | 0.0 | 0.0 | 0.0 | 0.0 | 0.0 | N/A | N/A | PM2_mod, PVS1_strong, PS4_strong | P | 12865758 |
|  |  | NM_004004.6  c.596C>T  p.Ser199Phe | Ms/Het | 1e05 | 9e06 | 0.0 | 5e04 | 0.0 | 0.0 | 0.0 | 0.0 | 0.0 | 1e05 | 0.9 | 0.9 | PM2_sup, PP3_strong, PS4_strong | P | 10376574 |
| Case 7 | *GJB2* | NM_004004.6  c.19C>T  p.Gln7* | Ns/Hom | 8e06 | 9e06 | 0.0 | 5e04 | 0.0 | 0.0 | 0.0 | 0.0 | 0.0 | 0.0 | N/A | N/A | PM2_mod, PVS1_strong, PS4_strong | P | 12865758 |
| Case 8 | *GJB2* | NM_004004.6  c.19C>T  p.Gln7* | Ns/Het | 8e06 | 9e06 | 0.0 | 5e04 | 0.0 | 0.0 | 0.0 | 0.0 | 0.0 | 0.0 | N/A | N/A | PM2_mod, PVS1_strong, PS4_strong | P | 12865758 |
|  |  | NM_004004.6  c.35delG  p.Gly12Valfs*2 | Fs/Het | 0.006 | 0.008 | 0.001 | 0.004 | 0.0 | 0.0008 | 0.009 | 0.003 | 0.008 | 0.007 | N/A | N/A | PVS1_strong, PS3_mod, PS4_strong | P | 9139825 |
| Case 9 | *GJB2* | NM_004004.6  c.19C>T  p.Gln7* | Ns/Het | 8e06 | 9e06 | 0.0 | 5e04 | 0.0 | 0.0 | 0.0 | 0.0 | 0.0 | 0.0 | N/A | N/A | PM2_mod, PVS1_strong, PS4_strong | P | 12865758 |
|  |  | NM_004004.6  c.35delG  p.Gly12Valfs*2 | Fs/Het | 0.006 | 0.008 | 0.001 | 0.004 | 0.0 | 0.0008 | 0.009 | 0.003 | 0.008 | 0.007 | N/A | N/A | PVS1_strong, PS3_mod, PS4_strong | P | 9139825 |
| Case 10 | *GJB2* | NM_004004.6  c.19C>T  p.Gln7* | Ns/Hom | 8e06 | 9e06 | 0.0 | 5e04 | 0.0 | 0.0 | 0.0 | 0.0 | 0.0 | 0.0 | N/A | N/A | PM2_mod, PVS1_strong, PS4_strong | P | 12865758 |
| Case 11 | *GJB2* | NM_004004.6  c.19C>T  p.Gln7* | Ns/Hom | 8e06 | 9e06 | 0.0 | 5e04 | 0.0 | 0.0 | 0.0 | 0.0 | 0.0 | 0.0 | N/A | N/A | PM2_mod, PVS1_strong, PS4_strong | P | 12865758 |
| Case 12 | *GJB2* | NM_004004.6  c.19C>T  p.Gln7* | Ns/Het | 8e06 | 9e06 | 0.0 | 5e04 | 0.0 | 0.0 | 0.0 | 0.0 | 0.0 | 0.0 | N/A | N/A | PM2_mod, PVS1_strong, PS4_strong | P | 12865758 |
|  |  | NM_004004.6  c.645delT  p.Arg216Aspfs*18 | Fs/Het | absent | absent | absent | absent | absent | absent | absent | absent | absent | absent | N/A | N/A | PM2_mod, PVS1_strong, PS4_sup | LP | 18758381 |
| Case 13 | *GJB2* | NM_004004.6  c.35delG  p.Gly12Valfs*2 | Fs/Hom | 0.006 | 0.008 | 0.001 | 0.004 | 0.0 | 0.0008 | 0.009 | 0.003 | 0.008 | 0.007 | N/A | N/A | PVS1_strong, PS3_mod, PS4_strong | P | 9139825 |
| Case 14 | *GJB2* | NM_004004.6  c.19C>T  p.Gln7* | Ns/Het | 8e06 | 9e06 | 0.0 | 5e04 | 0.0 | 0.0 | 0.0 | 0.0 | 0.0 | 0.0 | N/A | N/A | PM2_mod, PVS1_strong, PS4_strong | P | 12865758 |
|  |  | NM_004004.6  c.35delG  p.Gly12Valfs*2 | Fs/Het | 0.006 | 0.008 | 0.001 | 0.004 | 0.0 | 0.0008 | 0.009 | 0.003 | 0.008 | 0.007 | N/A | N/A | PVS1_strong, PS3_mod, PS4_strong | P | 9139825 |
| Case 15 | *GJB2* | NM_004004.6  c.427C>T  p.Arg143Trp | Ms/Hom | 0.0001 | 0.0004 | 0.0007 | 8e05 | 0.0001 | 9e05 | 0.0001 | 0.0 | 0.0001 | 0.0 | 0.9 | 0.9 | PM2_sup, PP3_strong, PS4_sup | LP | 9471561 |

All the frequencies were observed in gnomADv2.1.1, GRCh37/hg19

Zygosity (zyg), Missense (Ms), Nonsense (Ns), Frameshift (Fs), Splicing (Sp), Allele Frequency (af), African/African American (afr), Amish (ami), Admixed American (amr), Ashkenazi Jewish (asj), East Asian (eas), Finnish (fin), NonFinnish European (nfe), South Asian (sas), remaining individuals" (rmi), Not applicable (n/a), Rare Exome Variant Ensemble Learner (REVEL), AlphaMissense (AM), , supportive (sup), moderate (mod), Variant of Uncertain Significance (VUS), Likely Pathogenic (LP), Pathogenic (P), Homozygous (Hom), Heterozygous (Het), not available (N/A)
